# Recurrent Implantation Failure and the Gut–Endometrium Axis: A Pilot Study on Microbiota Crosstalk

**DOI:** 10.3390/jcm15197620

**Published:** 2026-10-01

**Authors:** Flora Caruso, Luigi Vigilante, Danilo Borrelli, Alessandra Gallo, Ida Strina, Attilio Di Spiezio Sardo, Maria Rosaria Fantuz, Giovanni Savarese

**Affiliations:** 1 Department of Public Health, School of Medicine, University of Naples “Federico II”, 80131 Naples, Italy; flora.caruso@hotmail.it (F.C.); danil.borrelli@gmail.com (D.B.); alessandra_gallo@hotmail.it (A.G.); ida.strina@unina.it (I.S.); attiliodispiezio@libero.it (A.D.S.S.); 2 AMES Centro Polidiagnostico Strumentale, 80013 Naples, Italy; rosy.fantuz@gmail.com (M.R.F.); giovanni.savarese@centroames.it (G.S.)

**Keywords:** implantation failure, microbiome, endometrium, gut, dysbiosis, infertility

## Abstract

**Objective**: We aim to assess gut and endometrial microbiota simultaneously in women with unexplained recurrent implantation failure (RIF) and explore the hypothesis of a gut–endometrium microbial axis. **Study design**: This monocentric observational pilot study included women aged 38 years or younger with a history of RIF. Paired fecal and endometrial samples were collected on the same day and analyzed by 16S rRNA gene sequencing. Relative abundances of major phyla and alpha-diversity indices were compared between compartments. **Results**: Twenty-one women were included. Chronic endometritis was documented through hysteroscopy in all cases. Gut dysbiosis was observed in 20/21 patients (95.2%), whereas endometrial dysbiosis was found in 16/21 (76.2%); crude agreement between compartments was 71.4% (Gwet’s AC1 0.62, 95% CI 0.25–0.90). The intestinal microbiota was dominated by Bacteroidetes (52.8% ± 14.9%) and Firmicutes (33.3% ± 11.0%), whereas the endometrial microbiota was enriched in Firmicutes (64.5% ± 33.5%) and Proteobacteria (30.9% ± 32.3%). Bacteroidetes were significantly more abundant in fecal than endometrial samples (*p* < 0.001), while Firmicutes were significantly enriched in the endometrium (*p* = 0.001). All alpha-diversity indices were significantly higher in feces, indicating greater microbial richness and complexity in the gut. **Conclusions**: In this pilot cohort of women with unexplained RIF, gut and endometrial dysbiosis frequently co-occurred. These findings support a potential gut–endometrium microbial axis and highlight the possible interplay between gut and endometrial microbiota in recurrent implantation failure. Further studies are warranted to better elucidate the clinical and biological implications of this relationship.

## 1. Introduction

Infertility is an increasing global public health issue, affecting millions of individuals and couples worldwide [1,2,3]. In parallel, the use of assisted reproductive technologies (ART) continues to rise, including in Italy, where medically assisted reproduction has contributed substantially to reproductive care in recent years [4,5]. Despite major advances in embryo culture, preimplantation genetic testing and endometrial assessment, implantation remains the limiting step of ART. Even after transfer of morphologically high-quality euploid embryos, implantation rates rarely exceed 50–60%, leaving a substantial proportion of patients without a clear explanation for failure [6,7].

Recurrent implantation failure (RIF) is among the most challenging conditions in reproductive medicine. The ESHRE good practice recommendations emphasize that RIF should be individualized according to patient context, while acknowledging the limited evidence supporting many currently used diagnostic and therapeutic approaches [8]. Persistent implantation failure has been associated with uterine, embryonic, endocrine, immunologic and infectious factors, yet a relevant fraction of cases remains unexplained despite standard evaluation [6,8,9].

Recent work has focused on the reproductive tract microbiome as a possible determinant of receptivity. Endometrial dysbiosis, usually characterized by reduced Lactobacillus dominance and enrichment of opportunistic taxa, has been associated with chronic endometritis, altered cytokine signaling and impaired implantation [10,11,12,13,14]. At the same time, growing evidence supports a gut-reproductive axis in which intestinal microbiota influences reproductive physiology through immune, metabolic and endocrine pathways [15,16,17,18,19,20]. The gut microbiota contributes to epithelial integrity, systemic inflammatory tone, short-chain fatty acid production and steroid hormone metabolism, all of which may affect the endometrium indirectly [15,16,17,18,19,20].

However, most studies have evaluated the gut and endometrial compartments separately. The present pilot study was designed to assess both compartments microbiota simultaneously in a highly selected cohort of young women with unexplained RIF, in order to explore their degree of concordance and to investigate whether endometrial dysbiosis may reflect a broader systemic microbial imbalance. The primary objective of this pilot study was to characterize the coexistence of gut and endometrial microbial alterations within the same women with unexplained recurrent implantation failure. Given its exploratory observational design and the absence of a control group, this study aimed to provide initial insights into the gut–endometrium microbial interplay and to identify potential microbial patterns warranting further investigation in controlled studies.

## 2. Materials and Methods

This monocentric observational pilot study was conducted at the IVF Unit of the Department of Gynecology and Obstetrics, University Federico II (Naples, Italy), between January and December 2025. Institutional Review Board and Ethics committee approval (Comitato Etico Campania 3 protocol code 129/2023, date of approval 6 December 2023) was obtained prior to the start of the research activities for this study, and all participants provided written informed consent before enrollment.

Patients referred to the hysteroscopy unit with RIF, meeting the following inclusion criteria, were enrolled: women aged 38 years or younger; diagnosis of RIF, defined according to the ESHRE good practice recommendations [8]; normal uterine cavity on hysteroscopic examination; and positivity to the ROME IV framework for bowel disorders [21]. Any minor anatomic abnormalities identified during diagnostic workup were corrected before enrolment.

Rome IV positivity was intentionally included as an eligibility criterion because the primary objective of this exploratory pilot study was to investigate the putative gut–endometrium axis in a clinically enriched population with disorders of gut–brain interaction (DGBIs). Accordingly, the presence of DGBIs in the entire cohort reflects the study selection criteria and should not be interpreted as an independent finding. Importantly, Rome IV criteria identify clinically defined DGBIs and were not used as a surrogate marker of intestinal dysbiosis, which was assessed independently through microbiota analysis.

Exclusion criteria were as follows: antibiotic use within 30 days before sampling; active pelvic infection; untreated endometrial pathology; and systemic inflammatory or autoimmune disease.

Relevant clinical information was recorded, including Body Mass Index (BMI), ART history, previous gastrointestinal, hormonal, anti-inflammatory and uterine treatments, and previous use of probiotics. All participants underwent a structured gastrointestinal assessment. Gastrointestinal symptoms were evaluated according to the Rome IV framework for bowel disorders [21], allowing classification into Irritable Bowel Syndrome (IBS) subtypes or severe constipation. Dietary patterns were assessed with specific attention to legume consumption and intake of ultra-processed foods, and lifestyle habits included regular physical activity.

Paired fecal and endometrial samples were obtained on the same day. Fecal material was collected in sterile containers and processed within two hours. Endometrial samples were obtained in the early proliferative phase (5th to 12th day of the menstrual cycle) using a sterile pipelle introduced under aseptic conditions to minimize contamination.

Following the procedure, the patients underwent hysteroscopy to assess the presence of chronic endometritis. Chronic endometritis (CE) was assessed through a combination of hysteroscopic morphological evaluation and endometrial biopsy. Hysteroscopy was performed using a 5 mm Bettocchi continuous-flow hysteroscope (Karl Storz, Tuttlingen, Germany) through a vaginoscopic approach, without analgesia or anesthesia, using normal saline as the distension medium. Following hysteroscopic assessment, an endometrial biopsy was obtained for histological evaluation and immunohistochemical identification of plasma cells using CD138 staining. CE was considered present when ≥3 plasma cells were identified per tissue section or ≥2 plasma cells per 10 high-power fields (HPFs).

Total DNA extraction was performed using standardized procedures validated for both high- and low-biomass samples. Taxonomic profiling was carried out by 16S rRNA gene sequencing. Relative abundances of the major phyla Firmicutes, Bacteroidetes, Proteobacteria and Actinobacteria were quantified. Microbial DNA was extracted using the QIAamp DNA Host-Free Microbiome Kit (QIAGEN, Hilden, Germany), according to the manufacturer’s instructions. Microbiota profiling was performed using the ARROWforNGS-Microbiota workflow, based on targeted amplification followed by next-generation sequencing (NGS). Primer Mix B was used to target the V3, V4, and V6 hypervariable regions of the bacterial 16S rRNA gene. Positive and negative amplification controls were included during library preparation. The positive control consisted of bacterial genomic DNA, whereas nuclease-free water was used as the negative control. Successful amplification was assessed by agarose gel electrophoresis. Sequencing was performed on an Illumina MiSeq platform. The mean sequencing depth was approximately 100,000 reads per fecal sample and 50,000 reads per endometrial sample. Sequencing data were processed using the dedicated ARROWforNGS bioinformatic software (MicrobAT version: 1.3.0), which generated the taxonomic profiling outputs.

Dysbiosis in both compartments was defined according to validated reference ranges derived from the previous literature and internal laboratory standards. Each microbiota was classified as physiologically balanced or dysbiotic based on deviations in composition and diversity.

Continuous variables are reported as mean ± standard deviation (SD) and median with interquartile range (IQR). Categorical variables are reported as absolute frequencies and percentages. Paired comparisons between fecal and endometrial microbiota were performed using the Wilcoxon signed-rank test. Agreement between gut and endometrial dysbiosis classifications was assessed on the 2 × 2 cross-tabulation. Because both prevalences exceeded 75%, rendering Cohen’s κ susceptible to the prevalence-dependent paradox, Gwet’s AC1 was computed as primary agreement index with 95% confidence intervals obtained by 10,000-iteration percentile bootstrap. Cohen’s κ with bootstrap 95% CI is also reported for transparency. A two-sided *p* value < 0.05 was considered statistically significant. Because of the exploratory nature of this pilot study, no formal a priori sample size calculation or statistical power analysis was performed. The sample size was therefore determined by the number of eligible patients enrolled during the predefined study period. Accordingly, all inferential analyses should be considered exploratory.

Statistical analyses were performed using Stata 19.0 (StataCorp, College Station, TX, USA).

## 3. Results

Twenty-one patients aged 38 years or younger were included. Chronic endometritis was documented in all the women (100%). Baseline clinical and gastrointestinal characteristics are summarized in Table 1.

Eighteen out of 21 patients (85.7%) were positive for Enterobacteriaceae. As expected from the ROME IV based eligibility criterion, all participants met criteria for a DGBI. Disorders of gut–brain interaction were present in the entire cohort, including severe constipation in 12 out of 21 women (57.1%), IBS with predominant Constipation (IBS-C) in 5 (23.8%), IBS with predominant Diarrhea (IBS-D) in 3 (14.3%) and Mixed IBS (IBS-M) in 1 (4.8%). Lifestyle-related factors were also frequent: 100% of patients reported junk-food consumption, only 9 of 21 (42.9%) consumed legumes weekly, and only 2 of 21 (9.5%) reported regular physical activity. Previous antibiotic exposure was recorded in 18 of 21 women (85.7%), and 8 of 21 (38.1%) reported NSAID use.

Gut dysbiosis was identified in 20 of 21 patients (95.2%), whereas endometrial dysbiosis was found in 16 of 21 (76.2%). Overall, 15 of 21 patients (71.4%) showed concomitant dysbiosis in both compartments. Crude observed agreement between gut and endometrial dysbiosis classifications was 71.4%. Because of the highly imbalanced marginal prevalences, Cohen’s κ was low (κ = 0.09), illustrating the prevalence-related limitation of this agreement measure in the present dataset. Gwet’s AC1, which is less sensitive to prevalence imbalance, was 0.62 (95% bootstrap CI: 0.25–0.90). The wide confidence interval reflects the small sample size and indicates substantial uncertainty in the estimated degree of agreement (Table 2).

Marked compositional differences emerged between compartments (Table 3). In fecal samples, the dominant phylum was Bacteroidetes (mean 52.8% ± 14.9%), followed by Firmicutes (33.3% ± 11.0%), Proteobacteria (10.4% ± 8.2%) and Actinobacteria (1.5% ± 2.6%). In endometrial samples, Firmicutes predominated (64.5% ± 33.5%), followed by Proteobacteria (30.9% ± 32.3%); Bacteroidetes were nearly absent (1.3% ± 1.7%), and Actinobacteria remained low (2.3% ± 2.7%). Bacteroidetes were significantly more abundant in feces than in the endometrium (*p* < 0.001), while Firmicutes were significantly enriched in the endometrium (*p* = 0.001). Proteobacteria showed a borderline higher abundance in the endometrium (*p* = 0.053), whereas Actinobacteria did not differ significantly (*p* = 0.242).

Alpha-diversity indices confirmed the greater ecological complexity of the intestinal microbiota (Table 3). The Shannon index was 3.8 ± 0.3 in fecal samples and 2.5 ± 0.8 in endometrial samples; the Chao1 index was 528.2 ± 90.7 and 359.5 ± 165.6, respectively. Similar differences were observed for the Gini–Simpson index (0.9 ± 0.0 vs. 0.8 ± 0.2) and Inverse Simpson index (22.5 ± 7.4 vs. 6.7 ± 5.2). All diversity comparisons were statistically significant (Shannon *p* < 0.001; Chao1 *p* = 0.004; Gini–Simpson *p* < 0.001; Inverse Simpson *p* < 0.001). These findings indicate that the endometrium represents a lower-diversity niche than the gut and may be more vulnerable to dysbiotic perturbations.

## 4. Discussion

In this pilot study of young women with unexplained recurrent implantation failure (RIF), gut dysbiosis was highly prevalent and frequently co-occurred with endometrial dysbiosis. Given the absence of a control group, these findings cannot establish whether the observed microbial alterations are specifically associated with RIF. Rather, the paired assessment of gut and endometrial microbiota provides descriptive evidence of their coexistence within the same highly selected cohort and is consistent with the hypothesis of an interconnected gut–endometrium microbial axis. The almost universal prevalence of gut dysbiosis, together with the high prevalence of endometrial dysbiosis and the observed degree of concordance between the two compartments, supports the rationale for investigating microbial alterations in RIF beyond the uterine environment alone. Nevertheless, the degree of formal agreement remains imprecisely estimated because of the small sample size and the markedly imbalanced prevalence of dysbiosis across the two compartments.

Importantly, the present study was not designed to compare women with and without RIF or to identify microbial features that are unique to RIF. Rather, the primary objective of this exploratory pilot study was to characterize the coexistence of gut and endometrial microbial alterations within the same women with unexplained RIF and disorders of gut–brain interaction (DGBI). Accordingly, our findings should be interpreted as descriptive and hypothesis-generating, providing a rationale for future controlled prospective studies aimed at determining whether specific microbial signatures are associated with RIF and whether alterations in the gut microbiota are mechanistically linked to the endometrial environment.

The endometrial microbiota in our cohort showed a relatively low-diversity profile dominated by Firmicutes and Proteobacteria, whereas the gut microbiota exhibited greater richness and was characterized predominantly by Bacteroidetes and Firmicutes. These findings are broadly consistent with previous studies suggesting that alterations in the endometrial microbiota may be associated with inflammatory signaling, impaired endometrial receptivity, and recurrent reproductive failure [10,11,12,13,14,22,23]. Previous investigations have frequently focused on reduced Lactobacillus dominance as a potential feature of an altered endometrial microbiota. However, genus-level abundance was not specifically assessed in the present analysis; therefore, no conclusions regarding Lactobacillus abundance or dominance can be drawn from our data.

In the gut compartment, the high prevalence of microbiologically defined dysbiosis observed in this clinically selected cohort occurred in the context of frequent previous antibiotic exposure and potentially unfavorable lifestyle patterns. Although these observations may provide relevant clinical context, the cross-sectional design and the absence of a control group prevent any inference regarding their contribution to the observed microbial alterations.

From a biological perspective, an interaction between the gut and endometrial microbial environments is plausible. The gut microbiota contributes to systemic immune regulation, epithelial barrier integrity, metabolite production, and enterohepatic hormone recirculation [15,16,17,18,19,20]. Persistent intestinal dysbiosis may promote low-grade systemic inflammation, alter cytokine signaling, and affect immune tolerance, potentially influencing distal mucosal sites, including the endometrium [15,17,19,20]. Within this framework, endometrial dysbiosis may represent either a local microbial alteration or a manifestation of a broader host–microbiome imbalance involving multiple mucosal compartments. The present findings cannot distinguish between these possibilities but support further investigation of the gut–endometrium axis in women with RIF.

Chronic endometritis should also be considered when interpreting these findings. Although chronic endometritis has been associated with alterations in the endometrial microbiota, the currently available evidence does not establish a reproducible microbiological signature beyond the frequently reported reduction in Lactobacillus dominance. Moreover, whether chronic endometritis represents a predominantly local condition or may occur within a broader host–microbiome dysregulation involving both the gut and endometrium has not been systematically investigated. Indeed, one of the motivations for the present pilot study was to begin exploring this unresolved question through the simultaneous assessment of gut and endometrial microbiota within the same patients. However, because chronic endometritis was present in all enrolled women, its independent contribution to the observed microbial alterations could not be evaluated.

This interpretation may be clinically relevant because women undergoing repeated ART failures are often exposed to multiple local and systemic interventions, including antibiotic treatments, hormonal stimulation, and intrauterine procedures. Although these interventions are intended to address factors potentially affecting implantation, repeated exposure may also influence microbiome composition and resilience, particularly at the intestinal level [16,17,18,24]. Whether persistent gut dysbiosis may contribute to an unfavorable endometrial environment, and whether correction of intestinal microbial alterations could influence endometrial receptivity, remain unanswered questions. Our findings therefore support a more integrated view of RIF, in which the endometrium may be considered within a broader host–microbiome network rather than as an isolated target organ.

Several limitations should be acknowledged. First, the study was monocentric and cross-sectional, and the sample size was small, limiting the precision and generalizability of the findings. Second, the absence of a fertile or otherwise appropriate control group precludes determining whether the observed microbial alterations are specifically associated with RIF or instead reflect characteristics of the broader population of women undergoing ART. Third, because chronic endometritis was present in all enrolled women, its potential contribution to the observed microbial profile could not be disentangled from that of RIF or other clinical factors.

In addition, specific genital hygiene practices were not systematically collected and therefore their potential contribution to endometrial microbial alterations could not be assessed. This represents an additional unmeasured potential confounder that should be considered in future studies. Low-biomass endometrial sampling also carries an inherent risk of contamination and may limit taxonomic resolution, which is particularly relevant when interpreting microbiome findings from the endometrial compartment [12,13,14].

Furthermore, the present exploratory analysis focused primarily on major bacterial phyla and alpha-diversity metrics and did not include predefined genus- or species-level differential abundance analyses. Consequently, taxon-specific conclusions, particularly regarding Lactobacillus in the endometrial compartment, cannot be drawn from the present dataset. In regard to statistical analysis, no correction for multiple comparisons was applied, as all eight paired tests were pre-specified descriptive endpoints within an exploratory pilot study. Accordingly, reported *p*-values should be interpreted with caution, as they are not adjusted for multiplicity and do not constitute confirmatory evidence.

Future adequately powered studies should incorporate predefined taxonomic analyses to determine whether specific microbial taxa or community structures are associated with RIF.

The study also lacked functional, metabolomic, and immunological measurements. Therefore, although the observed coexistence of gut and endometrial dysbiosis is biologically plausible within the framework of a gut–endometrium axis, the present data cannot directly demonstrate the immune or metabolic mechanisms potentially linking the two compartments. Integration of microbial composition with microbial metabolites, cytokine profiles, and other host-response markers will be essential to move from descriptive associations toward mechanistic understanding.

Despite these limitations, the paired assessment of gut and endometrial microbiota within the same women represents a strength of this study and provides preliminary data from a clinically challenging and understudied population. Larger, prospective, multicenter studies integrating microbiome, immunological, and metabolic profiling are warranted to determine whether gut microbial alterations are associated with endometrial dysfunction and, ultimately, whether restoration of gut eubiosis may influence endometrial receptivity and reproductive outcomes. To address these questions, a prospective observational study incorporating these complementary assessments has already been submitted for Ethical Committee approval by our group.

Finally, the near-universal prevalence of gut dysbiosis and the high prevalence of endometrial dysbiosis resulted in markedly imbalanced marginal distributions, limiting the interpretability of Cohen’s κ in this dataset. Gwet’s AC1, which is less sensitive to prevalence imbalance, suggested a relevant degree of agreement between the two compartments. However, the wide bootstrap confidence interval indicates substantial uncertainty around this estimate, and definitive conclusions regarding the strength of concordance cannot therefore be drawn. Replication in larger cohorts will be required to determine whether the observed degree of agreement is reproducible.

Overall, these findings provide preliminary descriptive evidence that gut and endometrial microbial alterations frequently coexist in women with unexplained RIF. While they do not establish an RIF-specific microbial signature or demonstrate a causal gut–endometrium relationship, they support the hypothesis that reproductive failure may occur within a broader host–microbiome context involving multiple mucosal compartments. This hypothesis warrants evaluation in larger controlled prospective studies with integrated taxonomic, functional, metabolic, and immunological profiling.

## 5. Conclusions

In conclusion, gut and endometrial dysbiosis frequently coexisted in women with unexplained RIF in this pilot cohort. These findings support the hypothesis of an interconnected gut–endometrium microbial axis, while the observational design and absence of a control group preclude conclusions regarding RIF-specific microbial alterations or causality. Larger, controlled prospective studies integrating microbiome, immunological, and metabolic profiling are warranted to further elucidate the relationship between these compartments and determine whether their microbial interplay may be relevant to implantation and reproductive outcomes.

## Figures and Tables

**Table 1:** Baseline clinical and gastrointestinal characteristics.

Variable	*n* (%)
Total sample	21
Chronic endometritis	21 (100.0)
Microbiology examinations	
*E. coli*/Enterobacteriaceae	18 (85.7)
Use of NSAIDs	8 (38.1)
Previous antibiotic exposure	18 (85.7)
Consumption of junk food	21 (100.0)
Weekly consumption of legumes	9 (42.9)
Regular physical activity	2 (9.5)
Gastrointestinal diagnosis	
IBS-D	3 (14.3)
IBS-C	5 (23.8)
IBS-M	1 (4.8)
Severe constipation	12 (57.1)

Values are reported as absolute frequencies and percentages. Microbiological results refer to conventional culture of stool samples.

**Table 2:** Prevalence of intestinal and endometrial dysbiosis.

		Endometrial Dysbiosis		Total
Gut dysbiosis		No	Yes	
	No	0	1	1
	Yes	5	15	20
Total		5	16	21

Observed agreement: 71.4%. Chance-expected agreement: 73.7%. Cohen’s κ = −0.09 (95% CI: −0.24 to 0.00). Gwet’s AC1 = 0.62 (95% CI: 0.25 to 0.90).

**Table 3:** Microbial composition and alpha-diversity in paired fecal and endometrial samples.

Variable	Feces	Endometrium	*p*-Value
Bacteroidetes (%)	52.8 (14.9) 58.2 [49.5–61.8]	1.3 (1.7) 0.3 [0.2–2.7]	<0.001
Firmicutes (%)	33.3 (11.0) 30.4 [28.4–32.9]	64.5 (33.5) 69.9 [45.4–97.1]	0.001
Proteobacteria (%)	10.4 (8.2) 9.0 [2.6–15.5]	30.9 (32.3) 25.9 [1.6–50.6]	0.053
Actinobacteria (%)	1.5 (2.6) 0.5 [0.3–1.6]	2.3 (2.7) 1.0 [0.4–3.6]	0.242
Shannon Index	3.8 (0.3) 3.9 [3.6–4.1]	2.5 (0.8) 2.7 [1.9–3.2]	<0.001
Chao1 Index	528.2 (90.7) 507.0 [453–606]	359.5 (165.6) 275.0 [250–406]	0.004
Gini–Simpson Index	0.9 (0.0) 1.0 [0.9–1.0]	0.8 (0.2) 0.8 [0.7–0.9]	<0.001
Inverse Simpson Index	22.5 (7.4) 24.0 [16.3–27.4]	6.7 (5.2) 4.7 [2.9–7.3]	<0.001

Data are reported as mean (SD) and median [25th–75th percentile].

## Data Availability

Data available on request to the corresponding author.

## Abbreviations

The following abbreviations are used in this manuscript:

ART	Assisted Reproductive Technology
RIF	Recurrent Implantation Failure
BMI	Body Mass Index
IBS	Irritable Bowel Syndrome
SD	Standard Deviation
IQR	Interquartile Range
IBS-C	IBS with predominant Constipation
IBS-D	IBS with predominant Diarrhea
IBS-M	Mixed IBS
DGBI	Disorder Of Gut–Brain Interaction
